# Cases of Influenza, with Remarks

**Published:** 1843-10

**Authors:** Solon Borland

**Affiliations:** Louisville


### Art. III.—*Cases of Influenza, with Remarks.* By Solon Borland, M.D., of Louisville.

Leaving to those who prefer, and are better qualified to perform it, the ambitious and "delightful task" of discussing the *philosophy* of epidemics in general; I propose to myself the more humble, if not more useful, duty of recording upon the pages of "The Western Journal," a few cases of the influenza, which has recently prevailed, not only in this city, but over a large portion of our country—ranging, generally, from the far north-east, to our south-western borders, and probably beyond them.

In doing this, I have no high expectation of adding any thing material to the stock of common-place knowledge, already so abundantly furnished, as well to the unprofessional observer as to the physician, by its almost universal prevalence, and its indiscriminate attack upon "all ages, sexes, and conditions" of the people; nor, yet, of presenting any views of theory or practice, remarkable for originality or value; but merely as a brief but faithful chronicle of the medical times, in relation to this particular epidemic, so far as it has passed under my own observation. I write from notes accurately made at the time—of observations carefully conducted.

Case I.—Mrs. W———, aged about 30 years, of small size and medium development, nervo-bilious temperament; the mother of several children—the youngest of which is now about eight months old. With previous children, has always menstruated regularly during lactation, as, also, with the last, until a little more than two months since, when the discharge failed at the regular period—with lumbar and hypogastric pains; and has thus remained; but her indisposition, on that score, was not such as to induce her to call in medical aid.

*Sunday, July* 16*th.*—I was called to see her this morning. On Friday night last, she felt the usual initiatory symptoms of the prevailing influenza: head-ache, sneezing, slight cough, and general muscular soreness. These symptoms have increased in severity up to this time. This is, also, her regular menstrual period. She now has very severe head-ache, her eyes are red and intolerant of light, skin dry and hot, face flushed, she has much hypogastric and lumbar pain, slight sore-throat, troublesome dry cough, sense of fatigue, great muscular soreness generally, and particularly about the chest; tongue thinly coated white, but full, moist, and otherwise natural; pulse 112, full and hard; bowels constipated.

I immediately bled her at the arm, to the amount of ℔iss; upon which there speedily followed slight nausea, considerable reduction of the force and frequency of the pulse, general perspiration, and abatement of pains and soreness. I then ordered ℞ pil. hydrag. gr. x.—pulv. ip. comp. gr. xij. ft. pil. no. iv. s. one pill to be given every two hours.

*Evening of the same day.*—She feels much better in all respects. I now prescribed, ℞ pulv. rhei gr. x., ext. colocynth. comp. gr. xx—ol. anis. gtt. iij.—ft. pil. no. vj. s. two of these pills to be given every hour.

*Monday morning, July 17th.*—There is no noticeable change in her condition since last evening. She has taken only one dose of the pills, and has had no motion of her bowels. I directed the remainder of the pills to be taken. *The blood drawn yesterday morning is deeply cupped, and covered with a very thick and tenacious buffy coat.*

*Tuesday morning, 18th.*—I do not visit her this morning; but her husband has called, and informs me that her bowels have been freely and easily moved—that her catamenia came on during the night, and that she is quite comfortable, with the exception of a slight cough, and some remains of muscular soreness about the chest when she coughs. I prescribed, ℞ vin. cohclici f℥ss., tinct. lobel. inflat. fℨij., syr. rhei simp. f℥iss., M. S. a teaspoonful to be given at intervals of two or three hours.

Her convalescence progressed satisfactorily.

Case II.—J. L———, a sailor, aged 26 years, of medium height, and spare habit, of a temperament not strongly marked, but inclining, I think, to the lymphatic. He has usually had very good health. His last voyage, made about eighteen months ago, from Norfolk to New Orleans, was a very tempestuous one, and, in consequence of severe exposure, he had a slight attack of articular rheumatism of the lower extremities; of which, however, he was soon relieved. About the first of this month, after bathing in the river, he had a slight attack of catarrh, of short continuance.

About a week ago, he felt the initiatory symptoms of influenza: head-ache, cough, general *malaise*, muscular soreness,

&c. These symptoms continued and increased in severity, for several days, when he was suddenly seized with a very acute pungent pain in the ball of the right foot, near the root of the great toe, attended with tenderness to the touch, and swelling of the part. This affection soon involved the whole foot, and, in regular and rapid succession, the ankle-joint, leg, knee, thigh, and hip, of that side; rendering the whole limb powerless, with constant acute pain, great heat, swelling, and exquisite tenderness—and, scattered here and there, over the more painful points, there appeared circumscribed red spots on the skin, about half an inch in diameter. Next day, the other lower extremity, and, in daily succession, each of the upper extremities, became affected in the same way—involving the shoulder-joints, and all the muscles of the chest; rendering the patient intirely helpless by his own efforts, and agonized by any attempt of others to move him; and his respiration painful and embarrassed.

*Wednesday afternoon, July* 19*th.*—I was called to see him this afternoon, and received the foregoing account: His condition is, generally, as above described. Besides, his skin is very hot, but moist; his tongue is red at the edges, and coated with tenacious mucus; there is no tenderness nor tension of abdomen, but he has had no motion of his bowels for several days; he has no appetite, but much thirst; and his urine is scant and high colored. His *pulse is feeble, small, exceedingly rapid, and intermittent.* (His intellect unimpaired). I prescribed an active cathartic, to be followed, after it had operated freely, by this mixture: ℞ vin. colchici f℥j., tinct. opi. f℥ss., ant. et potas. tart. gr. xij., syr. rhei simp. f℥iiss. misce et solve. S. a teaspoonful to be given him *every two hours.*

*Thursday morning, July* 20*th.*—The cathartic had operated well. The first dose of the mixture had vomited him, the second had produced some nausea, but was retained, and the succeeding doses had been borne well. His skin was not quite so hot, but still moist; his tongue not so red, and was fuller and moister, but darker, and more heavily coated. Thirst somewhat abated, but no return of appetite; pulse more voluminous, distinct, and regular, but not to be counted yet; there is no noticeable reduction of pain, swelling, &c. I directed the same mixture to be continued, in the same doses, but *every hour.*

*Evening of the same day.*—General condition not materially changed since morning, except, perhaps, the pains are rather more lancinating. The *pulse,* however, has undergone a very remarkable alteration: it can now be distinctly counted, *is* 112, *full, hard, and bounding; but quick, and not quite regular.* I now bled him at the arm, to the amount of ℔ij., when nausea, general and profuse perspiration, and softening of the pulse, with *regularity,* came on. The blood, when drawn, seemed unusually fluid, and was of a turbid, dirty, brown color. I directed the same mixture to be continued, as in the morning.

*Friday, noon, July* 21*st.*—He slept well last night, for the first time in more than a week; and is decidedly better, in all respects. His respiration is, comparatively, unembarrassed, and unattended with pain, and he can move his limbs a little; those first in the order of attack most freely. He passed urine copiously this morning, for the first time since his attack; and had a spontaneous motion of his bowels, of large quantity, and almost natural character. He has no undue thirst, and some desire for food; his tongue is without redness, full, and moist, and with a thick drab coating. The blood, drawn yesterday evening, presents a remarkable appearance: serum slightly in excess, and of a greenish color; coagulum exactly spherical, with a cup-like excavation at top, regularly formed, about three inches in diameter, and one inch and a half deep, lined with a buffy coat about half an inch thick, nearly, white, and nearly as firm and elastic as caoutchouc. I separated it from the clot, and preserved it in spirits, as a specimen.

I directed the same treatment to be continued.

*Evening of the same day.*—His general improvement has continued; but he has passed no urine since morning. I directed him to suspend the use of the mixture for the present, and take at one dose, the following powder: ℞ hydrarg. protochlorid. gr. x., potas. nitrat. gr. viij., pulv. rhei ℈j.

*Saturday morning, July 22d.*—The powder taken last night, has procured two good motions of the bowels, and abundant discharge of urine this morning. But he was restless and uncomfortable through the night, and does not feel so well as he did last evening. He has some increase of pain generally, and in the head, some cough, and a dry skin—pulse 100, quick, and hard. I bled him at the arm again, to the amount of ℔ij., when the pulse yielded, and the skin became moist. I now directed the mixture to be resumed, as before.

*Night of the same day, 9 o'clock.*—He is much improved in all respects, and is quite comfortable. The blood drawn this morning presents about the same appearance as that first drawn. I directed the same mixture to be continued, as before.

*Sunday, noon, July 23d.*—His improvement has progressed satisfactorily. I directed the same mixture to be continued; and, in addition, as an external application to the joints, twice a day, with gentle friction, the following liniment: ℞ Sem. dat. stramon. et sem. colchici contus. aa ʒij., ol. terebinth. fervent. f℥viij; infuse for three hours.

His convalescence, from this time, was rapid and satisfactory.

Case III.—Mrs. B———, aged 52 years, has for many years been in very delicate health, and subject to frequent attacks of severe illness, as pneumonia, dysentery, &c. She was attacked, about two weeks ago, with the usual initiatory symptoms of the prevailing influenza, as described in cases one and two. Received medical attention for about a week, without relief; then called in another physician, who has given her some attention, and now calls me in to see her. Dr. M. had bled her at the arm twice, once in small quantity, and once to the amount of ℥xij., and had her cupped over the upper front of the chest, with partial, but temporary relief. He had, also, purged her moderately with blue-mass. For several days past she has had excessive pain in the chest, particularly at the præcordia; and, three or four times a day, spasms of all the muscles of the chest, (and seeming to involve the *heart*) so as to suspend, almost intirely, for the time, from five to fifteen minutes, both respiration and circulation.

*Friday morning, July 21st.*—Found her in a spasm—*pulse very feeble, irregular, and intermittent*—respiration convulsive, and almost impracticable; almost unconscious of every thing, except a severe fixed pain at the præcordia. I made immediate application of Granville's strongest lotion, to the spine between the shoulders, with speedy partial relief; and as the spasm relaxed, I applied it over the seat of pain. In about ten minutes, the spasm passed off; but the pain persisted; and *the pulse rose full and powerful, but somewhat irregular, beating* 84 *in the minute.* I now bled her at the arm, to the amount of ℥xij; upon which the pulse became soft, slow, and more regular; and all her distressing symptoms were much relieved. As her bowels were constipated, I prescribed a pill, of the usual size, composed of equal portions of pil. hydrarg., pulv. rhei, and ext. colocynth. comp.; to be taken every hour, until free motion of the bowels should be procured.

*Evening of the same day.*—She has taken eight of the pills, without having any motion of the bowels; and has had three spasms during the day. I directed an *enema* of warm sweetened gruel.

*Saturday morning, 22d.*—The enema last evening, produced several very free, but painful motions of the bowels. After which, she felt much better, and passed the night in tolerable comfort; although she had several slight spasms during the night, and one this morning. Her pulse is quite active and strong, except during the spasms; and the pain at the præcordia continues. I now prescribed: ℞ Vin. colchici. f℥j., tinct. lobel. inflat. f℥ss., tinct. opi. camph. f℥iiss., ant. et potas. tart. gr. viij., misce et solve. S. give a teaspoonful of this mixture every hour.

*Evening of the same day.*—She has had no spasm, and but little pain, since this morning. The mixture has nauseated her greatly, and vomited her twice. Improvement in all other respects very decided; her pulse is greatly reduced in force, soft, compressible, and regular. I directed the mixture to be continued, but in smaller doses, and at longer intervals; also, use a warm pediluvium, and drink a tumbler of warm lemonade, containing sp. eth. nitros. f℥j., at bed time.

I should have stated before, that, throughout the attack, her urine has been scant and high colored, and voided with pain. She, also, has suffered for several years past, with internal hæmorrhoids, and during her present illness, they have been the cause of the pain attending the motion of her bowels.

*Sunday morning*, 23*d*.—After using the pediluvium, and drinking the lemonade, last night, she perspired freely, and passed urine more freely and easily than since the commencement of the attack. She has had no recurrence of spasm; and her pulse and skin feel natural. The mixture, which seems to have acted favorably in other respects, produces, now, constant and distressing nausea; although the dose has been greatly reduced, and the intervals of its administration greatly lengthened. In order to avoid this distressing nausea, if practicable, without discontinuing, yet, the antimonial and anodyne, I substituted for the mixture, this prescription: ℞ pil. hydrarg. gr. xij., ext. hyoscyam. gr. xxiv., ant. et potas. tart. gr. ij., ft. m. et in pil. div. no. xij. S. give one of the pills every two hours.

*Afternoon*.—She has taken two of the pills; but has been so much nauseated by them, that I am sent for. I find other symptoms as this morning. Directed half a pill to be taken at a dose.

*Night*.—Pills still nauseate, so that I abandon their use, and direct an enema at bed-time.

*Monday morning*, 24*th*.—Her bowels were gently, but efficiently moved, by the enema last night. She seems this morning to be so far relieved, as to require no further regular attendance.

From this time her convalescence was slow, but continuous.

4 *

Remarks.—The minuteness with which I have detailed the history, symptoms, and treatment, of the foregoing cases, leaves but little necessity for remarks to indicate the views of pathology and therapeutics, by which I was governed in their management, or to enable my readers to adopt views of their own. I make the general remark, however, that, in all the cases of the influenza seen by me, and particularly in the foregoing ones, the *inflammatory diathesis* was clearly and strongly marked; and that blood-letting was as clearly and as strongly indicated, as the prime and efficient remedial agent. True, there occurred a number of cases, as it will be found during the prevalence of all epidemics, no matter what the diathesis, so mild as to require no treatment at all. And it may be that most of the cases, however violent, would have yielded to other remedies, not so direct as blood-letting, but operating upon the same curative principle.

It were well for science and humanity, if the simple and comprehensive truth of these admissions could pierce the mental armor of those Polyphemi of the profession, who regard the most enlightened eclecticism as a medical Gallilee, out of which no good can come, and who would immolate whole hecatombs upon the altar of a *specific.* I have called this truth simple and comprehensive; and it is so. It pervades the universe, and is an element in all the varied operations of nature; and in all else, save the healing art, is as familiar, and as commonly admitted, as it is simple. It is, that there are various ways and means, by which the same end may be attained, the same thing accomplished. To deny this, in relation to any thing else than medicine, would justly subject a man to the epithet of idiot or lunatic. Yet, no department of human affairs is more pregnant with its value, or more demonstrative of its force, than medicine. His "cases of emergency" alone, furnish to the physician, who has any practice, conclusive proof of it; for there, by the use of means, which to the uninitiated seem the most *outré*, and unlike aught that had ever been applied to such purposes before, he has often been enabled to accomplish the most important objects, simply by knowing that the same *principle* of action runs through various and dissimilar means. He who has never overcome constipation of the bowels, by the use of the lancet, and, sometimes, even by a dose of opium, has, I apprehend, cultivated but a limited field of practical observation. It is told, in derision, of a Princess of England, (perhaps of her present gracious Majesty) that, upon learning that a portion of the liege subjects of that "free and happy country" were in a state of starvation, she exclaimed: "I would eat bread and cheese rather than starve!" And yet, this Princess, culpably ignorant as she was, of the condition of her people, was *wise*, in comparison with our medical exclusives, and sticklers for specifics. *She did* know, intuitively perhaps, that the same principle of nutriment was to be found, alike, in the dainty luxuries on which she had fed, and in the very dissimilar articles of homely bread and cheese; while *they*—philosophers, too!—*cannot be taught* that when we would reduce inflammation by *depletion*, that principle may be brought into action by means, either of bleeding, purging, puking, or sweating, &c.; the peculiar circumstances of each case, rendering the one or other of these remedies the more eligible means. I have not run into this digresssion without design. It has not been, however, so much to find fault with the views of others, as to set forth, upon its true ground, my own preference of particular remedies; and, in this instance, of blood-letting. I have said that in this epidemic, I considered blood-letting the prime and efficient remedy. But, after what I have said about exclusiveness, I cannot be understood as contending that it was indispensable to, or alone capable of affording, relief. By no means. I do insist, however, that in *all* inflammatory diseases, the abstraction of blood being the most *direct*, should be the *prime*, and will prove to be the most *efficient* curative means. This is a general therapeutic principle, which I believe to be as sound and immutable as truth itself; modified, however, but only in its *application*, by the variety of cases.

While in the spirit of digression, I must pay my respects to those practitioners, some of whom it has been my misfortune to meet, who seem to be afflicted with *Hæmatophobia*, (if I may coin a word). These gentlemen, according to my observation, may be divided into two classes, on account of the two kinds of influence under which they respectively, yet alike, manifest their "holy horror" of blood-letting. The one class seem to me to oppose blood-letting for the very reason which mainly induces me to prefer it: because it is a *direct* remedy, in its application and results: because it can be understood and appreciated by the common mind, and is not enshrouded in mystery. Mark—with what unction these same gentlemen will roll an *anti-bilious pill*—measure, with nicest exactitude, an *alterative mixture*—and adjust the scales, "to the ninth part of a hair," in weighing out a *deobstruent powder!* tasting and smelling, the while; and looking as solemn, and as wise withal, as the sage bird of night! Might I venture on a comparison, I would call them a sort of *mental prism*—incapable of receiving into themselves, or of transmitting, the rays of light in *straight* lines, as they come down upon them from the great sun of nature's truth; but *refract* and pervert them to unwise, and sometimes, alas! to unholy ends. To them, it is folly in the extreme, to come boldly out into the light; but the profundity of wisdom, to burrow, like a mole, along the dark and devious track of indirection. The other class, who are certainly the more respectable—because the more honest, seem to oppose blood-letting, because, forsooth, they remember to have heard that, in holy-writ, it is declared—"Whoso shedeth man's blood, by man shall his blood be shed." A literal interpretation of the text, truly! and worthy of the days of witch-craft. It can be matched only by a kindred interpretation of another portion of scripture, once perpetrated by a young lady of my acquaintance. This young lady, at church one day, was dismayed to hear that it was forbidden to use *feathers* for personal adornment. Terror-stricken at the *enormous* sin she had been so ignorantly committing, she hurried home, and incontinently emptying out the contents of the *pillow*, with which she had exaggerated her fair proportions, refilled the sack with a "bale of *cotton*," instead of feathers. And, thus, by a literal *ruse*, was my fair friend enabled, without any "compunctious visitings," and with due modesty, to wear both her conscience and her *bustle*. These instances speak well, doubtless, for the conscientious scruples, and simple piety of these enlightened practitioners of medicine, and of this ingenious young lady; but they make, at the same time, a lamentable, and a ludicrous, exposure of their want of discrimination. These persons seem utterly incapable of distinguishing between the naked act itself, and the motive and object which prompt to its commission. With them, it is a crime to draw blood; whether to destroy, or to save life, is no matter.

It is a dogma of a noted charlatan, whose followers are, even now, scattered over this land, that "the blood is the life of man;" and that, therefore, to shed a *drop* of it, is, *pro tanto*, to be a murderer. "'Tis true, and, pity 'tis, 'tis true!" that this absurdity is not limited to "the blind followers of the blind;" but attaches, with disgraceful and fatal tenacity, to some who claim, by right of *parchment* too, a joint-tenancy in the domain of medical science.

Again, there are others, who are driven from the abstraction of blood, by fear of the goblin-sprite *debility!* which, with the damps of death distilling from its wings, has too long hovered over the morbid world. To these last, I would suggest that, in a vast number of cases, debility is but a *simulation*, and, consequently, is then most readily removed by blood-letting; while, even in those cases where it is really present, certain modifications of this remedy are, still, the most efficient means of relief; for, let it ever be borne in mind, that *debility*, however certainly present and prominent, in any case, is always a *symptom*—a consequence, and never a *cause*—or essential character of disease. And to all, whether the lovers of *indirection*, the tender of conscience, the followers of the charlatan, or the slaves of their own timidity, I would say without the fear of successful contration, that for every *single* case wherein they can show me the evil consequences of blood-letting (though I will not deny that evil has followed it), I will point to hundreds who, for want of its timely use, "have died of medicable wounds."

I will return now from these, perhaps unprofitable, digressions; and conclude this paper with a few remarks more directly bearing upon the subject in hand, and particularly upon the cases I have reported.

I repeat, that in all the cases of the influenza which I saw, the inflammatory diathesis was strongly marked. In some cases, as is found in all epidemics, the attack was so slight, that nature was competent to her own relief; but even in these, the tendency was to inflammatory engorgements in the most exposed and susceptible tissues; and analogy warrants the belief that depletion of some kind, particularly blood-letting, would have hastened the cure. In more violent cases, the inflammatory action was more decided; but yielded readily to depletion, as bleeding and gentle purgatives. In my own case, which was among the first that occurred in the city, and quite threatening from the rapidity and violence with which the inflammatory symptoms were developed, the disease was arrested almost at once, by one very free bleeding (nearly ℔iv.), and a gentle cathartic. In several similar cases, but little more was required. But, in the cases of greatest violence, such as I have reported, and particularly the last two, other and more persevering treatment was demanded. It will be seen that in them, I not only bled, but purged, and used other active remedies, of known power to reduce inflammatory action. In rheumatic inflammations, and other affections wearing their livery, I had been long accustomed to use, with success, tartarized antimony combined with opium. Finding, in these cases of influenza, a strong family likeness of rheumatism, I resorted, with confidence, to this potent remedy. At my own suggestion, I should not have used the colchicum or lobelia, either singly or in combination. I was induced to do so on the advice of my talented friend Dr. John R. Buck, who, at the time, was using the particular combination I prescribed, with marked advantage, in many cases of influenza, and analogous affections. He was led to use it himself, and to recommend it to me, in view of the strongly manifest rheumatic and bronchitic symptoms coexisting in all the cases. In his cases, Dr. B. is satisfied of its efficacy; and his judgment is to be relied on. But, where I used it, I used, at the same time, other well tried remedies which, heretofore, had not failed me in time of need; and I am not prepared to say how much of the cure must be ascribed to the new, and how much to the old remedies. It is but right, however, to add, that in a few cases of a very mild character, where I was requested to prescribe for a cough, and slight muscular soreness, I prescribed the combination alluded to, on account of its neat and eligible form, and was gratified to find it intirely successful.

Of the particular cases reported, I will remark: That case number one, was, as regards the symptoms peculiar to influenza, but little beyond the average of severity. The main suffering of the patient was caused by the complication of painful amenorrhœa. Case number two, was among the severest, if it were not the very severest, that occurred in the city. I have never seen any case of disease where the inflammatory action ran so high, and, at the same time, involved so extensively the fibrous system. From the neck, downwards, no muscle, ligament, or cartilage, seemed exempt. I am satisfied that the diaphragm, and the *heart*, were seriously implicated. To this last, I attribute the peculiar character of the pulse. It will be remembered, also, that the blood in this case, presented a remarkable appearance: the form of the clot, and the great amount of *fibrous crust.* If the buffy-coat be, as is generally held, the sign and *measure* of inflammatory action, then this may well stand at the head of inflammatory cases. I think, however, I have observed, that, *cæteris paribus*, the fibrous crust, or buffy coat, of drawn blood, is always most abundant in inflammations of fibrous tissues, particularly in rheumatism. I have never observed it in the simple phlegmasiæ of mucous membranes. Does this accord with the observations of others?

Case number three, was one of great severity. There were circumstances, however, well calculated to exacerbate all its symptoms beyond the ordinary range of the epidemic. The feeble health, and excitable condition of the patient, particularly of the nervous and circulatory systems, owing to previous severe and exhausting attacks of various and complicated diseases, rendered her peculiarly liable to suffer exceedingly, and even to sink, under any present attack, of even ordinary severity. Besides, the case had been either neglected, or treated very timidly in the beginning. So that, we found not only a bad original subject, but one in which disease had been allowed, almost unmolested, to take a firm hold, even of the very heart-strings. This last is literally true; for that the heart, in this case, was seriously involved, no one can doubt who will study the case. Indeed, when I first saw her, such was the extraordinary vigor of the pulse, that, taking into account the age, feeble frame, and delicate health of the patient—particularly as she had been long subject to pain in the chest, occasional difficult respiration, and palpitations of the heart, I suspected the existence of chronic hypertrophy of the heart, upon which had supervened the acute *carditis.* The result of the case, however, removed the suspicion of hypertrophy.

It was my purpose to have prepared the foregoing cases, &c., for the September number of this Journal; but I have deferred it until now, in the hope of being able to compare notes, usefully, with some of my correspondents, both north-east, and south-west, of this place; but, in the main, I have been disappointed. My esteemed and intelligent friend Dr. J. Robert Ward, of Maryland, alone, furnishes me information which I can turn to useful account. In his letter of 22d August, he gives me a very clear and interesting account of the influenza, as it prevailed at, and in the vicinity of, his residence—*Clear Spring.* The symptoms, as described by Dr. W., correspond very accurately with what I have observed and described. A tolerably close correspondence, too, may be observed in the treatment pursued by each. In his own words:—"I have used the lancet, salts and magnesia, salts and soda, and followed them up with tartar-emetic, and tr. of opium." He further observes, that: "when more active cathartics were used, a troublesome diarrhœa was the result." As to the pathology, Dr. W. expresses views not so accordant with mine. At first, he believed, with me, that the disease was decidedly inflammatory; judging both from the symptoms and the effect of remedies. Subsequent observation and reflection, have shaken, if not changed this opinion; and chiefly because the disease was of such short continuance (rarely continuing, with any severity, "more than thirty hours") and yielded so readily to comparatively mild remedies. He inclines to the opinion that the seeming violence of the symptoms, and the great suffering of the patients, resulted from exalted sensibility, which might be characterized as a *nervous erethism.* Had I not, already, occupied too much space, I think I could easily show that this, although plausible, is not the proper view. But whether his or my pathology be true, he considers the proper treatment was the one pursued. He deems the colchicum and lobelia particularly adapted to his view. Without controverting any of his opinions, I conclude by tendering him my respectful and sincere thanks for his kindness and courtesy.

*Louisville,* Sept. 22d, 1843.

